# Meta-Analysis Comparing Watchman^TM^ and Amplatzer Devices for Stroke Prevention in Atrial Fibrillation

**DOI:** 10.3389/fcvm.2020.00089

**Published:** 2020-06-22

**Authors:** Indranill Basu Ray, Dibbendhu Khanra, Sumit Shah, Sudhanva Char, Xiaoming Jia, Wilson Lam, Nilesh Mathuria, Mehdi Razavi, Bhavna Jain, Dhanunjaya Lakkireddy, Saibal Kar, Andrea Natale, Adedayo Adeboye, John Lynn Jefferies, Sripal Bangalore, Samuel Asirvatham, Mohammad Saeed

**Affiliations:** ^1^Memphis VA Hospital, Memphis, TN, United States; ^2^University of Memphis, Memphis, TN, United States; ^3^All India Institute of Medical Sciences (AIIMS), Rishikesh, India; ^4^University of Arkansas for Medical Sciences, Little Rock, AR, United States; ^5^Biostatistics, Life University, Marietta, GA, United States; ^6^Baylor College of Medicine, Houston, TX, United States; ^7^Texas Heart Institute, Houston, TX, United States; ^8^University of Kansas Medical Center, Kansas City, KS, United States; ^9^Cedars-Sinai Medical Center, Los Angeles, CA, United States; ^10^Texas Cardiac Arrhythmia Institute at St. David's Medical Center, Austin, TX, United States; ^11^University of Tennessee Health Science Center, Memphis, TN, United States; ^12^New York University School of Medicine, New York, NY, United States; ^13^Mayo Clinic College of Medicine, Rochester, MN, United States

**Keywords:** atrial fibrillation, stroke, left atrial appendage closure, Amplatzer Cardiac Plug, Amplatzer Amulet, Watchman^TM^ device

## Abstract

**Background:** For patients with atrial fibrillation who are at high risk for bleeding or who cannot tolerate oral anticoagulation, left atrial appendage (LAA) closure represents an alternative therapy for reducing risk for thromboembolic events.

**Objectives:** To compare the efficacy and safety of the Amplatzer and Watchman^TM^ LAA closure devices.

**Methods:** A meta-analysis was performed of studies comparing the safety and efficacy outcomes of the two devices. The Newcastle-Ottawa Scale was used to appraise study quality.

**Results:** Six studies encompassing 614 patients were included in the meta-analysis. Overall event rates were low for both devices. No significant differences between the devices were found in safety outcomes (i.e., pericardial effusion, cardiac tamponade, device embolization, air embolism, and vascular complications) or in the rates of all-cause mortality, cardiac death, stroke/transient ischemic attack, or device-related thrombosis. The total bleeding rate was significantly lower in the Watchman^TM^ group (Log OR = −0.90; 95% CI = −1.76 to −0.04; *p* = 0.04), yet no significant differences was found when the bleeding rate was categorized into major and minor bleeding. Total peridevice leakage rate and insignificant peridevice leakage rate were significantly higher in the Watchman^TM^ group (Log OR = 1.32; 95% CI = 0.76 to 1.87; *p* < 0.01 and Log OR = 1.11; 95% CI = 0.50 to 1.72; *p* < 0.01, respectively). However, significant peridevice leakages were similar in both the devices.

**Conclusions:** The LAA closure devices had low complication rates and low event rates. Efficacy and safety were similar between the systems, except for a higher percentage of insignificant peridevice leakages in the Watchman^TM^ group. A randomized controlled trial comparing both devices is underway, which may provide more insight on the safety and efficacy outcomes comparison of the devices.

## Introduction

Left atrial appendage (LAA) closure is an alternative means of reducing the risk for stroke in patients with atrial fibrillation (AF) who cannot tolerate long-term oral anticoagulation (1–4), as the vast majority of thrombus formation in patients with AF occurs in the LAA (5, 6). Several LAA closure systems have been developed, including the Watchman^TM^ Left Atrial Appendage Closure Device (Boston Scientific, Marlborough, Massachusetts) and the Amplatzer^TM^ Cardiac Plug and Amulet devices (St. Jude Medical-Abbott, St. Paul, Minnesota) (7). Of these, the Watchman^TM^ is the most studied device and the only device approved by the U.S. Food and Drug Administration (FDA) for LAA closure. FDA approval was based on data from two multicenter randomized controlled trials comparing the Watchman^TM^ and warfarin: the PROTECT AF (Watchman^TM^ Left Atrial Appendage System for Embolic PROTECTion in Patients With Atrial Fibrillation) trial (8) and the PREVAIL (Prospective Randomized Evaluation of the Watchman^TM^ LAA Closure Device in Patients With Atrial Fibrillation vs. Long-Term Warfarin Therapy) trial (9). Notably, in the PREVAIL trial, non-inferiority was established for ischemic stroke prevention but not for overall efficacy (a composite of stroke, systemic emboli, and cardiovascular or unexplained death) (9). A 2-year follow-up study of data from the prospective, multicenter, multinational EWOLUTION (Evaluating Real-Life Clinical Outcomes in Atrial Fibrillation Patients Receiving the Watchman^TM^ Left Atrial Appendage Closure Technology) registry showed that patients with AF who received a Watchman^TM^ device had consistently low rates of stroke and non-procedural bleeding (10). Despite their limited use in the United States, the Amplatzer LAA closure devices are popular in the rest of the world. Many observational studies showing favorable safety and efficacy outcomes have been published (11–20). Several cohort studies have directly compared the safety and efficacy of the Amplatzer cardiac plug or amulet vs. the Watchman^TM^ device (21–26). However, these studies were limited in sample size and therefore we conducted a meta-analysis of published data that directly compared the Amplatzer and Watchman^TM^ devices, focusing primarily on efficacy and safety outcomes (27).

## Methods

### Search Strategy

A systematic review of existing literature was performed to search for literature prior to July 2019. Two physician-reviewers (DK and SS) queried PubMed, EMBASE, and the Cochrane Central Register of Controlled Trials (CENTRAL) databases for published literature, using the search terms Watchman^TM^, Amplatzer Cardiac Plug, Amplatzer Amulet, left atrial appendage occlusion, left atrial appendage closure, and combinations of these keywords. Additional literature was sought by searching for references of eligible articles. Any discrepancies were resolved by a third reviewer (IBR).

### Study Selection

**Figure 1:**
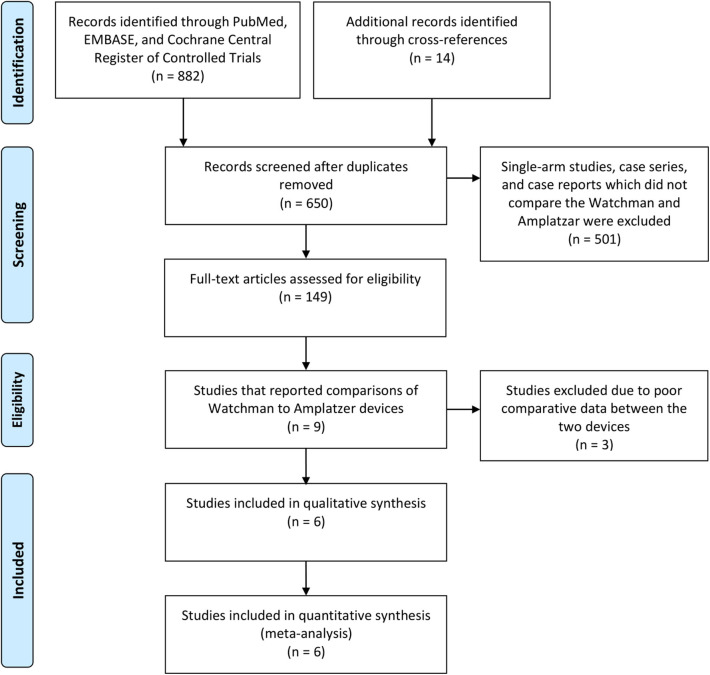
PRISMA Flow Diagram: Schematic of Systematic Literature Search.

For the meta-analysis, we selected studies that directly compared the Amplatzer and Watchman^TM^ devices, provided periprocedural and at least 90 days long-term efficacy data (similar follow up duration for both the devices), and had a sample size of at least 10 (Figure 1). Studies that involved both the Amplatzer and Watchman^TM^ devices but did not report comparative outcomes data for each device were excluded (28–30). Single-arm studies, case reports, case series, and cohort studies that had fewer than 10 patients or that did not present adequate safety/efficacy outcomes data also were excluded.

The Newcastle-Ottawa Scale was used to assess whether the studies included in the meta-analysis were of good or poor quality (31). Good quality is indicated by 3–4 points in the selection domain, 1–2 points in the comparability domain, and 2–3 points in the outcome domain (for an overall rating of 6–9 points) (Supplementary Table 1).

### Data Extraction

Data on baseline characteristics and safety and efficacy outcomes for each device were extracted from each of the selected studies and entered into a Microsoft Excel spreadsheet by authors DK and SS. Baseline characteristics include the total number of participants and implantation success rate for individual studies of age, sex, previous stroke, and CHA_2_DS_2_-VASc (congestive heart failure, hypertension, age, diabetes, stroke/TIA, and VAScular disease) score (for determining stroke risk and prophylaxis). Also included were HAS-BLED (hypertension, abnormal renal/liver function, stroke, bleeding, labile international normalized ratio, elderly, and drugs/alcohol) score (for determining risk for major bleeding associated with oral anticoagulation), mean follow-up periods, follow up duration for post-procedural trans-esophageal echocardiography (TEE), and size for assessment of significant peridevice leakages. Safety outcomes included periprocedural complications such as cardiac tamponade, pericardial effusion, device embolization, air embolism, and vascular complications. Long-term efficacy outcomes included all deaths, cardiac death, stroke or TIA, bleeding, peridevice leakage, and device-related thrombosis. The study outcome definitions from the individual studies were used for the above outcomes.

### Data Analysis

To compare the safety and efficacy outcomes of the two devices, a hypergeometric-normal model to approximate the exact likelihood was used, as the number of events in each study is small relative to the group and included many zero events (32). To negate the small study effect, Log OR with 95% confidence intervals (CIs) was calculated, and that was back-transformed to predict exponential OR along with 95% confidence intervals (CIs) using R software (33).

## Results

Six studies with a total of 342 patients in the Watchman^TM^ group and 274 (Table 1) patients in the Amplatzer group were included in the meta-analysis (Figure 1) (21–26). All of the studies scored 7 points on the Newcastle-Ottawa Scale (Supplementary Table 1), indicating good quality. Descriptive comparison of baseline characteristics from the six studies used in the meta-analysis is shown in Table 1. Follow-up durations for post-procedural trans-esophageal echocardiography (TEE) were different across the studies and have been summarized in Table 1. The definitions of significant peridevice leakages were also variable across the studies and have been summarized in Table 1. The implantation success rate was high for both the Amplatzer (98.2%) and the Watchman^TM^ (96.8%) devices. The mean procedural time was 55.1 ± 6.2 (minutes ± standard deviation) for Watchman^TM^ and 59.3 ± 11 for Amplatzer, while the mean fluoroscopy time was 9.9 ± 3.8 for the Watchman^TM^ and 12 ± 4.8 for the Amplatzer. The *I*^2^ statistic for heterogeneity was 0% for most outcomes, with the highest statistic being 55.74%, signifying consistency of results. A statistical comparison of baseline characteristics, procedural outcomes, and safety and efficacy outcomes is shown in Table 2. In most studies, the background anticoagulants regimens included single antiplatelet therapies for patients for whom oral anticoagulants were contraindicated. Post-operatively, most of these patients were discharged on dual antiplatelet treatment.

**Table 1:** Descriptive comparison of baseline characteristics.

**References**	**N (Procedural Success)**		**Age (year) Mean** **±** **SD**		**Female (%)**		**Previous stroke/TIA (%)**		**CHA**_****2****_**DS**_****2****_**-VASc Mean** **±** **SD**		**HAS-BLED Mean** **±** **SD**		**Follow-up period (months)**	**TEE follow up for DRT**	**Significant peridevice leakage**
	**A**	**W**	**A**	**W**	**A**	**W**	**A**	**W**	**A**	**W**	**A**	**W**			
Chun et al. (21)	40 (100%)	40 (95%)	76 ± 9	76 ± 9	16	18	12	6	4.5 ± 1.8	4.1 ± 1.5	3.1 ± 1.2	3.1 ± 1.1	12.0	6 weeks	>5 mm
Cruz-Gonzalez et al. (22)	21 (100%)	10 (100%)	77 ± 9	77 ± 5	7	5	-	-	3.3 ± 0.5	3.7 ± 0.8	3.9 ± 0.9	4.7 ± 1.3	3.0	3 months	>3 mm
Gafoor et al. (25)	27 (92.6%)	26 (92.3%)	84 ± 3	83 ± 3	8	12	10	8	5.0 ± 1.5	5.3 ± 1.4	–	–	12.0	6 months	Not reported
Figini et al. (24)	99 (100%)	66 (100%)	72 ± 9	72 ± 8	28	28	29	19	4.0 ± 1.7	3.8 ± 1.6	3.7 ± 1.5	3.4 ± 1.3	15	W: 84 days (52–128)^*^ A: 81 days (57–188)^*^	>3 mm
Kim et al. (26)	52 (96.1%)	46 (100%)	65 ± 10	66 ± 9	18	19	22	20	3.6 ± 1.6	4.1 ± 1.7	2.7 ± 1.3	2.8 ± 1.2	21.9	6 weeks	>3 mm
Fastner et al. (28)	35 (97.1%)	154 (96.1%)	77 ± 9	75 ± 2	13	49	8	44	4.0 ± 1.4	4.5 ± 0.1	3.7 ± 1.0	3.6 ± 0.2	6	6 months	>5 mm

* *TEE follow up day in median (minimum to maximum)*.

*CHA_2_DS_2_-VASc, Congestive heart failure, Hypertension, Age, Diabetes, Stroke/TIA, VASscular disease risk score; DRT, Device-related thrombus; HAS-BLED, Hypertension, Abnormal renal/liver function, Stroke, Bleeding, Labile international normalized ratio, Elderly, Drugs/alcohol risk score; TEE, Trans-esophageal echocardiography*.

**Table 2:** Statistical comparison of safety and efficacy outcomes.

**Variables**	**Log OR [95% CI]**	***P*-value**	***I*^**2**^**	**Tau^**2**^**	**Q**	**Predicted OR [95% CI]**
**Safety outcomes**						
Cardiac tamponade	0.20 [−1.28 to 1.68]	0.79	0%	0	0.80	1.22 [0.28 to 5.35]
Pericardial effusion	0.54 [−0.39 to 1.47]	0.26	0%	0	0.61	1.71 [0.68 to 4.34]
Device embolization	−0.85 [−2.68 to 0.98]	0.36	0%	0	0.74	0.43 [0.07 to 2.68]
Air embolism	−0.57 [−2.13 to 0.99]	0.47	0%	0	0.34	0.56 [0.12 to 2.69]
Vascular complications	0.38 [−0.85 to 1.61]	0.55	0%	0	1.98	1.46 [0.43 to 4.99]
**Efficacy outcomes**						
All-cause mortality	−0.70 [−1.70 to 0.29]	0.17	0%	0	3.19	0.50 [0.18 to 1.34]
Cardiac Death	−0.70 [−2.44 to 1.04]	0.43	0%	0	0.40	0.50 [0.09 to 2.83]
Stroke/ TIA	0.30 [−1.06 to 1.66]	0.66	0%	0	0.59	1.35 [0.35 to 5.28]
Total bleeding	**−0.90 [−1.76 to** **−0.04]**	**0.04**	0%	0	–	**0.41 [0.17 to 0.96]**
Major bleeding	−0.93 [−2.03 to 0.16]	0.09	4.27%	0.06	2.26	0.39 [0.13 to 1.17]
Minor bleeding	−0.59 [−2.04 to 0.86]	0.43	0%	0	0.22	0.56 [0.13 to 2.36]
Total Peridevice leakage	**1.32 [0.76 to 1.87]**	** <0.01**	0%	0	**1.87**	**3.74 [2.15 to 6.52]**
Significant Peridevice leakage	1.11 [−0.60 to 2.82]	0.20	55.74%	1.63	7.05	3.04 [0.15 to 62.94]
Insignificant Peridevice leakage	**1.11 [0.50 to 1.72]**	** <0.01**	0%	0	**1.76**	**3.05 [1.66 to 5.60]**
Device-related thrombosis	0.60 [−0.55 to 1.75]	0.30	0%	0	1.88	1.83 [0.58 to 5.77]

*Boldface values indicate significant at p < 0.05*.

*CHA_2_DS_2_-VASc, Congestive heart failure, Hypertension, Age, Diabetes, Stroke/TIA, VASscular disease risk score; CI, confidence interval; HAS-BLED, Hypertension, Abnormal renal/liver function, Stroke, Bleeding, Labile international normalized ratio, Elderly, Drugs/alcohol risk score; OR, Odds ratio; Q, Cochrane's Q value; TIA, transient ischemic attack*.

### Safety Outcomes

Overall, periprocedural complication rates were low in both groups (Table 2). The only vascular complications were local groin hematoma and pseudoaneurysm formation. The meta-analysis found no significant differences in the safety outcome measures, which included cardiac tamponade, pericardial effusion, device embolization, air embolism, and vascular complications.

### Efficacy Outcomes

The mean follow-up period to assess the outcomes ranged from 3 to 22 months. The overall complication event rates were low in both groups (Table 2). No significant differences were observed in all-cause mortality, cardiac death, and stroke or TIA occurrences. The total bleeding rate was significantly lower in the Watchman^TM^ group (Log OR = −0.90; 95% CI = −1.76 to −0.04; *p* = 0.04). However, no significant differences were observed when major bleeding and minor bleeding outcomes were compared.

Device-related thrombosis and peridevice leakage data were taken from transesophageal echocardiography results obtained at the first follow-up visit after the LAA closure procedure (Table 1). However, because definition of peridevice leakage varied across the studies, comparisons were made for significant, insignificant, and total peridevice leakage rates. The rates of total peridevice leakage were significantly higher in the Watchman^TM^ group (Log OR = 1.32; 95% CI = 0.76 to 1.87; *p* < 0.01). Significant peridevice leakage rate was similar among the Watchman^TM^ and the Amplatzer group, whereas insignificant peridevice leakage rate was significantly higher in the Watchman^TM^ group (Log OR = 1.11; 95% CI = 0.50 to 1.72; *p* < 0.01). Device-related thrombosis rate did not differ significantly between the Watchman^TM^ and Amplatzer groups.

## Discussion

In this study, we performed a systematic review and meta-analysis to compare the two most popular LAA closure devices worldwide: the Watchman^TM^ Left Atrial Appendage Closure Device and the Amplatzer Cardiac Plug and Amulet devices. Of these devices, only the Watchman^TM^ has been approved in the United States for stroke prophylaxis in patients with AF. FDA approval was granted in response to two randomized controlled trials (PROTECT AF and PREVAIL) that demonstrated the Watchman's^TM^ non-inferiority to anticoagulant in preventing strokes (8, 9, 34). However, although ample efficacy and safety data on the Amplatzer devices are available from European and Asian reports (11–20), the devices have not been broadly adopted in the United States.

This spurred the FDA to recommend a head-to-head trial comparing Watchman^TM^ and Amplatzer devices in patients undergoing LAA closure (34). To this end, a randomized clinical trial of the Watchman^TM^ device and the Amplatzer Amulet is now underway in Switzerland. The primary endpoints of the SWISS-APERO trial (NCT03399851) are composites of many of the outcome measures evaluated in our study, including a safety composite (procedure-related complications, all-cause death, and major bleeding through 12 months), an efficacy composite (ischemic stroke and systemic embolism through 18 months), and a mechanism-of-action endpoint (device closure at 45 days, as evaluated by transesophageal echocardiography). The trial is expected to begin providing results by no later than 2021.

### Safety Outcomes

In terms of real-world data, many of the Watchman^TM^ safety outcomes were derived from the PROTECT AF (8, 35) and PREVAIL (9) trials and their subsequent registries, whereas Amplatzer data came from smaller cohort studies. Reddy et al. (34) observed that, over time, increased user experience with the Watchman^TM^ resulted in a trend toward fewer periprocedural complications. Our meta-analysis showed that, overall, both devices had relatively low complication rates. We found no differences in the rates of periprocedural complications with regard to pericardial effusion, cardiac tamponade, major bleeding, vascular complications, device emboli, or air embolization; nonetheless, the strength and generalizability of these results is tempered by the small sample size and low number of adverse events.

### Efficacy Outcomes

The meta-analysis showed no significant difference between the two devices with respect to overall mortality, cardiac deaths, and stroke or TIA occurrences. Even though the total bleeding rate was higher in the Amplatzer group, major and minor bleeding rates were not significantly different for the devices. Low chi^2^ value in the presence of a tau^2^ value of 0 signifies the consistency of results and is unlikely to be tampered with by the variable follow-up period. It should be noted that post-operative antithrombotic therapy varied for the devices and for each study. Manufacturer recommendations regarding post-procedural antithrombotic protocols are different for the Amplatzer vs. the Watchman^TM^. After Amplatzer implantation, patients are usually started on dual antiplatelet therapy and then transitioned to aspirin monotherapy; conversely, after Watchman^TM^ implantation, patients typically receive oral anticoagulation for 45 days before transitioning to dual antiplatelet therapy and eventually aspirin monotherapy. This may be one of the possible explanations for the significant difference in total bleeding incidences.

Significant peridevice leakage was defined differently in the six studies included in the analyses. Total peridevice leakages were significantly more frequent in the Amplatzer group. However, significant peridevice leakages were similar among both the devices which matches the finding of the EWOLUTION registry, where the rate of significant leaks in Watchman^TM^ devices was <1% (10). Low chi^2^ value in the presence of tau^2^, value of 0, signifies the consistency of results that are unlikely to be tampered with by the variable follow-up period. Device-related thrombus (DRT) events were similar for both the devices despite different follow-up durations across the studies. Not all patients who followed up underwent TEE (21–26). However, in a large meta-analysis combining 66 studies, there was no difference in DRT rates between the Amplatzer and Watchman^TM^ devices (3.6 vs. 3.1%, *p* = 0.24) (36).

The device design might also contribute to the discrepancy in peridevice leakage rates between the two closure systems. The Amplatzer devices comprise a lobe and a disk, connected by a central waist. The self-expanding lobe engages the LAA, while the disk covers the orifice. The Watchman^TM^ is a parachute-shaped device consisting of a self-expanding frame with a permeable polyester fabric cover that accommodates the LAA. Several studies have examined mechanisms of peridevice leakage, which represents suboptimal engagement of the device with the LAA (37, 38). Post-operative imaging studies have shown that Watchman^TM^ leaks stem primarily from gaps between the device and the LAA ostium, whereas Amplatzer leaks typically involve off-axis alignment of the lobe portion with respect to the LAA neck (37). However, Wolfrum et al. (38) found no correlation between periprocedural complications and the positioning of the Amplatzer device.

Notably, a study of the anatomical impact of the devices in canine models showed more complete neo-endocardialization of the Watchman^TM^ device surface vs. the Amplatzer after 28 days. This was postulated to result from the Amplatzer disk's extension outside of the LAA orifice, which may affect surrounding structures and delay healing (39). These anatomical differences do not appear to significantly affect either device's overall efficacy in stroke prevention, and subsequent studies of the effect of incomplete LAA closure and peridevice leakage found no significant increases in long-term adverse events (40).

### Limitations

The number of prospective studies included in the meta-analysis were limited. Of the six studies, two were prospective non-randomized studies, four were retrospective studies, and no randomized controlled trials were available. Although funnel plots were generated to appraise efficacy and safety outcomes, because our meta-analysis included fewer than 10 studies, the data were probably too scant to detect true asymmetry from chance, and thus no definitive conclusions could be drawn.

## Conclusions

These devices have different implant techniques. The Amplatzer Device includes a lobe with a covering disk (connected by a waist) and the Watchman^TM^ device includes a self-expanding frame covered by a fabric. Post-implantation anti-coagulation regimens are also different for the devices. The Watchman^TM^ typically has a longer duration of both anti-coagulants and dual anti-platelet therapy post-implantation in comparison to the Amplatzer. However, the results of this study reveal an overall low and similar complication rate for both devices. The total bleeding rates were higher in the Amplatzer group (but not major or minor bleeding), and other efficacy endpoints were similar in both groups, except for a higher percentage of insignificant peridevice leakages in the Watchman^TM^ group. Our observations were limited by the small number of available studies. An FDA-mandated randomized controlled trial directly comparing these two popular devices is currently underway, and its results may provide more comparative information about the safety and efficacy of the devices.

## Perspectives

**Competency in Medical Knowledge 1:** Patients with atrial fibrillation (AF) are at a relatively higher risk of developing transient ischemic attack (TIA) or stroke. However, antithrombotic therapy may not be a feasible option for such patients if they are at a higher risk of bleeding or cannot tolerate the therapy.

**Competency in Medical Knowledge 1:** Contemporary left atrial appendage (LAA) closure devices (The Watchman^TM^ and the Amplatzer) provide an alternative option to mechanically occlude the LAA, thereby eliminating the need for antithrombotic therapy in patients with higher bleeding rates or who cannot tolerate antithrombotic therapy.

**Competency in Patient Care:** Given the high implantation success frequency and low frequency of adverse outcomes, LAA closure is a promising avenue for the specific cohort of patients to minimize the risk of TIA or strokes. However, regular long-term follow-ups are recommended for both devices to ensure their functionality and to record the safety and efficacy outcomes.

**Competency in Interpersonal and Communication Skills:** Even though the newer anticoagulants have proved superior in providing effective anticoagulation and lower internal bleeding rates as compared to warfarin, indolent bleeding is still a critical issue for certain populations. Since the devices work mechanically and have a low risk of adverse outcomes, alternative possible treatment options should be discussed with eligible candidates.

**Translational Outlook 1:** Even though only the Watchman^TM^ is approved in the United States, the Amplatzer is approved in other countries and has been successful in demonstrating low rates of adverse events in other countries. However, further research and long-term comparison of safety and efficacy outcomes of both devices is required due to their structural dissimilarities.

**Translational Outlook 2:** Additional prospective clinical trials are needed to evaluate the long-term outcomes of both devices.

## Data Availability Statement

All datasets generated for this study are included in the article/Supplementary Material.

## Author Contributions

IB conceptualized and reviewed the analyses. DK and SS performed the literature review, analyses, and drafted the manuscript. SC, XJ, WL, and BJ also contributed to drafting the manuscript and critical review of the text. Critical review of the manuscript and inputs regarding the analyses, results, and interpretation of the results were received from NM, MR, DL, SK, AN, AA, JJ, SB, SA, and MS. All authors contributed to the article and approved the submitted version.

## Conflict of Interest

SK receives research grants from, and is a consultant for, Boston Scientific, Abbott Vascular, Gore Medical. The remaining authors declare that the research was conducted in the absence of any commercial or financial relationships that could be construed as a potential conflict of interest.

## Abbreviations

AF: atrial fibrillation
CI: confidence interval
FDA: US Food and Drug Administration
LAA: left atrial appendage
OR: odds ratio
TIA: transient ischemic attack.
