# Supplementary material for: Meta-Analysis Comparing WatchmanTM and Amplatzer Devices for Stroke Prevention in Atrial Fibrillation
Source: Front Cardiovasc Med. 2020 Jun 22;7:89. doi: 10.3389/fcvm.2020.00089 (PMC7322993; doi:10.3389/fcvm.2020.00089)
Supplement: Supplementary file 1 [file Table_1.docx]

**ONLINE SUPPLEEMNT**

**TABLE 1** Newcastle-Ottawa Scale Scores of Studies Included in the Meta-Analysis Comparing the Amplatzer and Watchman Devices

| Study | Selection | Comparability | Outcome | Total |
| --- | --- | --- | --- | --- |
| Chun et al. 2013 | 3 | 1 | 3 | 7 |
| Cruz-Gonzalez et al. 2014 | 3 | 1 | 3 | 7 |
| Gafoor et al. 2014 | 3 | 1 | 3 | 7 |
| Figini et al. 2017 | 3 | 1 | 3 | 7 |
| Kim et al. 2016 | 3 | 1 | 3 | 7 |
| Fastner et al. 2018 | 3 | 1 | 3 | 7 |
